# Alpelisib-Induced Diabetic Ketoacidosis in a Patient With Metastatic Breast Cancer

**DOI:** 10.7759/cureus.20817

**Published:** 2021-12-29

**Authors:** Jawaher Al Zeyoudi, Imad El Kebbi, Fatima Al Zaabi, Shahrukh Hashmi

**Affiliations:** 1 Internal Medicine, Sheikh Khalifa Medical City, Abu Dhabi, ARE; 2 Endocrinology, Sheikh Shakhbout Medical City, Abu Dhabi, ARE; 3 Oncology, Sheikh Shakhbout Medical City, Sheikh Khalifa Medical City, Abu Dhabi, ARE

**Keywords:** oncology, hyperglycemia, alpelisib, metastatic breast cancer, diabetic ketoacidosis

## Abstract

We report the case of a 45-year-old woman with metastatic breast cancer who started treatment with alpelisib nine days before developing diabetic ketoacidosis (DKA). At the time of DKA diagnosis, blood tests showed a capillary blood glucose of 30 mmol/L, serum carbon dioxide level of 11 mmol/L, an anion gap of 25 mEq/L, and a glycated hemoglobin A1C (HbA1c) level of 6.4% (50 mmol/mol). Her HbA1C on admission was 5.6% (38 mmol/mol). Capillary blood glucose levels improved upon discontinuation of alpelisib and returned to baseline four days after drug discontinuation. DKA is a rare but serious adverse effect of alpelisib. Patients on this medication should be closely monitored for hyperglycemia and DKA. Further studies are needed to help identify patients at risk of hyperglycemia and DKA.

## Introduction

Breast cancer remains the most common cancer in women globally [1] and the cancer with the highest incidence among women in the Middle East [2]. In particular, the Health Authority of Abu Dhabi (HAAD) reported that in 2014, breast cancer was the most common malignancy diagnosed in Abu Dhabi (United Arab Emirates), accounting for 20.3% of new cancer cases [3]. In addition, breast cancer was responsible for 12.2% of all cancer deaths in the Emirates in 2015 and was the leading cause of cancer death among women [3].

Around 70% of breast cancers are hormone receptor (HR)-positive and human epidermal growth factor receptor 2 (HER2)-negative [4]. Of those patients, 40% have a PI3KCA mutation [5]. The prognostic and therapeutic implications of these mutations have been shown in many studies [6].

In 2019, the FDA approved the use of alpelisib in addition to fulvestrant for the treatment of postmenopausal women and men with (HR)-positive, (HER2)-negative, PIK3CA-mutated breast cancer. However, the use of alpelisib has been associated with many adverse effects, with hyperglycemia, rash, and gastrointestinal adverse effects being the most common [7]. Hyperglycemia was reported in 63.7% of patients treated with alpelisib in phase III SOLAR-1 trial, and diabetic ketoacidosis (DKA) was reported in 0.4% of patients (one patient out of 284) [7]. A few reports have been published since then documenting new-onset DKA in patients with a history of prediabetes or no prior history of abnormal glycemia [8-11].

## Case presentation

We present the case of a 45-year-old woman who presented to our emergency department with dyspnea, diffuse abdominal pain, and abdominal distention for one week. Her past medical history was significant for metastatic breast cancer (HR-positive and HER2-negative), refractory to multiple regimens of chemotherapy. She had no prior personal or family history of diabetes mellitus. Her home medications were exemestane, everolimus, ergocalciferol, and melatonin. On admission, the patient was alert and oriented; her blood pressure was 104/65 mmHg, and her heart rate was 81 beats per minute. Her weight was 68 kilograms, and her height was 160 cm. Physical examination showed a distended abdomen with no guarding or rebound. She was found to have ascites on ultrasound, and ascites fluid analysis was consistent with transudative fluid (serum ascitic albumin gradient was 1.8 g/dL). The peritoneal fluid culture showed no growth, and the cytology analysis showed no malignant cells.

Due to the progression of her metastatic breast cancer, her oncologist stopped her home medications, exemestane and everolimus, and started her on alpelisib 300 mg daily, in addition to one dose of intramuscular fulvestrant 500 mg. At the same time, she was started on metformin 1000 mg twice daily. On admission, her hemoglobin A1C (HbA1c) was 5.6% (38 mmol/mol). Her capillary blood glucose reading started to increase on day 3 after starting alpelisib, reaching 28-30 mmol/L. Due to worsening acute kidney injury, metformin was suspended, and she was started on insulin glargine at bedtime and insulin aspart three times daily in addition to correction doses of insulin aspart. Despite titrating her insulin regimen according to her blood glucose, she continued to have hyperglycemia ranging between 15 and 20 mmol/L but remained hemodynamically stable.

On day nine after starting alpelisib, the patient complained of shortness of breath and diffuse abdominal pain. Her vital signs were within acceptable range except for a respiratory rate of 25 breaths/min. She was alert and fully oriented. Laboratory examination showed a capillary blood glucose of 39 mmol/L, serum potassium of 5 mmol/L, serum sodium of 123 mmol/L, serum chloride of 90 mmol/L, serum carbon dioxide of 11 mmol/L, albumin-corrected anion gap of 25 mEq/L, and serum ketones of 2 mmol/L consistent with diabetic ketoacidosis. Venous blood gas showed a pH of 7.27, partial pressure of carbon dioxide (pCO2) of 28.5 mmHg, and a lactate level of 2.4 mmol/L. Repeat glycosylated HbA1c was 6.4% (50 mmol/mol). Alpelisib was discontinued, and the patient was started on the hospital DKA protocol, which included aggressive hydration with normal saline and an intravenous regular insulin infusion. Her anion gap closed, and her glucose levels continued to improve. Intravenous insulin was transitioned to a subcutaneous insulin regimen within 24 hours of initiation.

**Figure 1:**
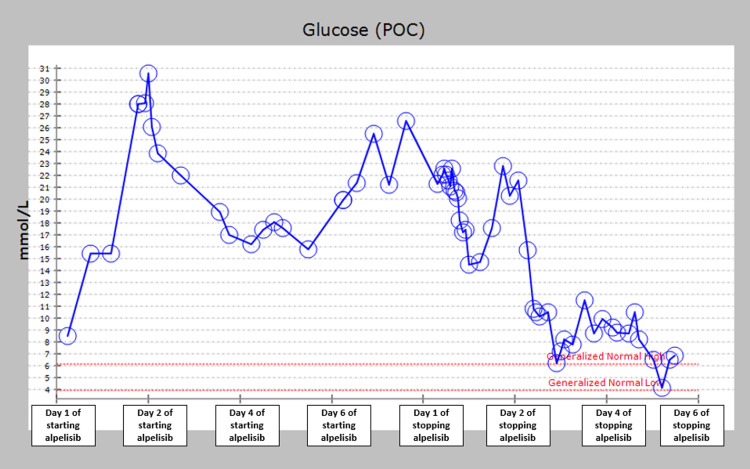
Graph demonstrating capillary blood glucose (CBG) from the start of alpelisib initiation and throughout the hospital stay

Five days after alpelisib was discontinued, the patient’s serum glucose started to improve, and her subcutaneous insulin was tapered and finally discontinued eight days after alpelisib was stopped. Blood glucose readings remained normal until her discharge (Figure 1).

## Discussion

Alpelisib is an inhibitor of the alpha subunit of p110 alpha of phosphatidylinositol 3-kinase (PI3K), an enzyme that plays a key role in the PI3K/AKT kinase pathway [12]. This pathway plays a major role in many aspects of cell life, including cell survival and maturation, and glucose regulation. It has been reported that about 30% of patients with malignant diseases were found to have mutations in that enzyme, leading to over-activation and dysregulation of cell processes. Therefore, targeting this pathway is expected to carry anti-neoplastic effects and to become a promising precision therapy for patients with these mutations.

Even though alpelisib in combination with fulvestrant has been approved for use in patients with (HR)-positive and (HER2)-negative metastatic breast cancer, supporting evidence for the presence of PIK3CA mutation is needed before starting therapy. Studies have shown that patients treated with alpelisib in combination with fulvestrant achieve a response rate of 26.5%. However, adverse effects are common and include hyperglycemia, nausea, vomiting, loss of appetite, and diarrhea [7].

Hyperglycemia is one of the most common adverse effects associated with alpelisib and appears to be the result of a reduction in insulin sensitivity. It has been reported to occur in 51%-65% of patients taking alpelisib in clinical trials and was the most common grade 3 or 4 adverse effect leading to dose reduction or discontinuation [13]. There are many different ways by which this medication causes insulin resistance. The genes encoding many glycolytic enzymes are under transcriptional control of the PI3K/AKT pathway. Activation of AKT stimulates glucose uptake and metabolism [14]. Therefore, drug-induced inhibition of PI3K/AKT reduces glucose uptake, which leads to hyperglycemia and stimulation of insulin secretion. Glucose transport capacity, glycogen synthesis, and glycolysis have also been reported to be reduced by close to 40% by alpelisib.

In the SOLAR-1 trial, the majority of patients, 51.5%, had grade 3 hyperglycemia. Hyperosmolar hyperglycemic state and diabetic ketoacidosis were extremely rare. Grade 4 hyperglycemia with blood glucose > 500 mg/dl occurred in 3.9% of trial patients, and 0.4% developed DKA. To reduce these adverse effects, the inclusion criteria of phase III clinical trial SOLAR-1 were amended to include patients with a fasting plasma glucose at or below 140 mg/dl and HbA1C below 6.5% [13]. The safety of alpelisib in patients with type 1 and uncontrolled type 2 diabetes has not been determined as these patients were excluded [7].

We found four cases of alpelisib-induced DKA reported in the literature [8-11]. The oldest patient was 73 years old, and the youngest was 49 years old. One patient had prediabetes, whereas the others had no history of diabetes. The period between starting alpelisib and admission for DKA ranged between 11 days and three months. Patients exhibited elements of insulin resistance and had to be treated with insulin, frequently in high dosage. Hyperglycemia improved promptly with the discontinuation of alpelisib.

The SOLAR-1 trial study protocol was amended to include the management guidelines for hyperglycemia; mild elevations in fasting plasma glucose (≤160 mg/dl) do not require alpelisib dose modifications, but starting metformin 500 mg once daily is recommended [13]. The dose can be raised by 500 mg at weekly intervals if tolerated, up to a maximum dose of 2000 mg daily. For fasting plasma glucose readings between 160 and 250 mg/dl, alpelisib can be maintained at the same dose unless hyperglycemia persists for more than 21 days despite the glucose-lowering medications. If the metformin dose is already maximized and fasting plasma glucose readings do not improve, adding another insulin-sensitizer agent like pioglitazone should be considered.

In patients who develop fasting plasma glucose >250 mg/dl, treatment with alpelisib should be stopped for one to two days until hyperglycemia improves. Treatment with insulin may be used as needed. Blood glucose tends to go back to baseline in the majority of patients with drug interruption, given the short half-life of alpelisib, and long-term treatment with insulin is rarely necessary [13].

## Conclusions

Alpelisib is a relatively new and promising drug for patients with advanced breast cancer, which carries hope for patients with other types of solid cancers as well. It is, however, associated with significant side effects, with hyperglycemia being fairly common, and DKA occurring rarely. Checking fasting plasma glucose and HbA1C levels is required before treatment initiation, in addition to monitoring glycemia during therapy. Patients need to be educated to recognize the symptoms of hyperglycemia (e.g., polyuria, polydipsia, or polyphagia) before taking this medication. Close monitoring is particularly needed for patients with prediabetes or at the risk of diabetes due to excess adiposity, a strong family history of diabetes, or ethnicity that is known to be at risk for diabetes, such as people of Middle Eastern, South Asian, or Latino descent. Further studies are needed to help identify additional risk factors for hyperglycemia and DKA so that early dietary, lifestyle, and/or pharmacologic interventions are undertaken.
